# Reproductive toxicity of a nano-insecticide (chlorpyrifos) on male albino mice

**DOI:** 10.1038/s41598-026-56438-0

**Published:** 2026-06-10

**Authors:** Marwa Abdeltawab Ahmed, Ahmad Ali Kandeel, Wessam Salim Tawfik

**Affiliations:** ¹Zoology Department, Faculty of Science, Fayoum University, Fayoum, Egypt

**Keywords:** Nano-emulsion, Chlorpyrifos, Male Mice, Reproductive Toxicity, Reproductive Performance, Biochemistry, Physiology, Zoology

## Abstract

Chlorpyrifos (CPF) is a commonly used organophosphorus insecticide in agriculture, and it is linked to reproductive toxicity in males by disrupting the endocrine system. Most previous studies focus on its traditional form, neglecting its nanoform, which may pose a greater risk due to enhanced bioavailability. Our study evaluated the reproductive toxicity of a nano-emulsion of CPF (CPF-NE) in male albino mice. Mice were divided into four groups (*n* = 10 per group): a control and three treated groups receiving CPF-NE via oral gavage at doses of 3.79, 7.58, and 15.16 mg/kg body weight daily for 35 days. We assessed gonadosomatic index (GSI), reproductive performance (mating success and fertility rate), sperm parameters, DNA fragmentation, hormonal levels (testosterone, FSH, LH), total antioxidant capacity (TAC), malondialdehyde (MDA), and catalase (CAT). Post-treatment testes were examined histologically. Results showed that CPF-NE significantly decreased GSI (from 0.66 ± 0.02 in controls to 0.31 ± 0.02 in high-dose, *p* < 0.001), reproductive performance (fertility index dropped to zero at high dose), sperm viability (from 74 ± 1.6% to 43.1 ± 1.7%, *p* < 0.001), and testosterone (from 4.2 ± 0.3 ng/mL to 1.8 ± 0.2 ng/mL, *p* < 0.001), while increasing FSH, LH, MDA, and DNA fragmentation. Histopathological findings indicated reduced tubular diameters, decreased epithelial heights, and disrupted testicular structure. In conclusion, CPF-NE adversely affects male fertility by increasing oxidative stress and DNA damage.

## Introduction

Organophosphates (OPs) rank among the most commonly utilized insecticides worldwide, appreciated for their pest control efficacy. However, their widespread application has sparked concerns regarding potential reproductive toxicities, mainly in male fertility. Studies suggest that exposure to OPs can result in notable negative impacts on sperm quality and overall reproductive health^1^. Traditional agricultural strategies often rely heavily on chemical pesticides, which adversely affect animals and humans while also diminishing soil fertility. As a result, nanotechnology has been proposed to provide eco-friendly and effective solutions for managing insect pests in agriculture without harming nature. However, the drawbacks of nanotechnology can occur in both occupational and residential environments^2^.

Nanomaterial-based formulations offer advantages such as improved efficacy due to increased surface area, higher solubility, and systemic activity due to smaller particle size, higher mobility, and lower toxicity by eliminating organic solvents compared to traditional formulations^3^. However, ingestion of nano-pesticides could be problematic in both occupational and residential settings^4^. According to Iftikhar et al.^5^, nanomaterials can easily pass through the blood-testis barrier and, once accumulated in the testis, negatively affect spermatogenesis.

Chlorpyrifos (CPF) is a traditional organophosphorus insecticide approved as a broad-spectrum insecticide in 1965. It has been frequently employed to manage a wide range of pests in agriculture and animal farms^6^. Exposure of laboratory animals to CPF causes a variety of effects, including hepatic and testicular damage^7,8^. CPF and other OPs primarily inhibit acetylcholinesterase (AChE) activity by phosphorylation. AChE hydrolyzes the neurotransmitter acetylcholine, removing it from the synapse and stopping impulse transmission^9^. Additionally, exposure to CPF can raise intracellular reactive oxygen species (ROS) levels, potentially causing male reproductive harm through oxidative stress in sperm^10^.

Recently, nanoformulations of CPF have been used successfully to control the red palm weevil both in the field and in the laboratory^11^. However, few studies have discussed the toxic effects of nanomaterials, especially the nanoform of CPF, on male reproductive indices and performance. Therefore, the present study was designed to evaluate the potential reproductive toxicity of nano-formulated chlorpyrifos (CPF-NE) in male albino mice, with particular emphasis on hormonal balance, oxidative stress, spermatogenic integrity, and histopathological changes.

## Materials and methods

### Chemicals

Chlorpyrifos (CPF) was obtained from the National Research Institute (Giza, Egypt) with 97% purity in crystal form. It was converted into a 10% nano-emulsion (CPF-NE) at Nanofab Technology Center, 6th October City, Egypt, following^12^. The nano-emulsion was characterized by dynamic light scattering (DLS) using a Zetasizer Nano ZS (Malvern Instruments, UK), which confirmed an average droplet size of 35 ± 15 nm and a polydispersity index (PDI) of 0.26, indicating uniform size distribution. The zeta potential was measured as -32.5 ± 4.2 mV, suggesting good physical stability of the formulation. CPF-NE was diluted in corn oil to achieve the desired doses. The control group received corn oil only.

### Experimental Animals

Male albino mice (Mus musculus), aged 10 weeks and weighing 30 ± 2 g, were obtained from VACSERA (Helwan, Egypt). Animals were adapted for two weeks under controlled conditions (12-hour light/dark cycle) with free access to food and water. All procedures were approved by the Scientific and Ethical Committee of Fayoum University **(Approval No AEC2449-a).**

## Experimental Design

### Determination of LD50

The LD50 of CPF-NE was evaluated by the graphical method of Miller and Tainter^13^. Sixty adult male mice weighing 30 ± 2 g were randomly divided into six groups (*n* = 10 each). Different oral doses were administered to each group based on body weight. Mortality rates were recorded over 24 h. The percentage of mortality was plotted against the logarithm of the applied dose after applying the correction formulas:

0% dead animals = 100 × (0.25/n).

100% dead animals = 100 × [(n-0.25)/n]

where (n) is the number of animals per group. The dose corresponding to 50% mortality was determined as the acute oral median lethal dose (75.6 mg/kg body weight).

### Doses and treatment

Animals were divided into four groups (*n* = 10 each). They were orally gavaged with CPF-NE daily for 35 days (one spermatogenic cycle)^14^:

Group I: control (corn oil).

Group II: low dose (3.79 mg/kg, 1/20 LD50).

Group III: mid dose (7.58 mg/kg, 1/10 LD50).

Group IV: high dose (15.16 mg/kg, 1/5 LD50).

Clinical symptoms were documented daily. After 35 days of exposure, all mice were euthanized by cervical dislocation, and necropsied to collect blood and reproductive organs (testes and epididymis) for further analysis. Carcasses and residual tissues were disposed of in accordance with Fayoum University’s biosafety and animal facility rules.

### Reproductive performance study

Treated males were mated with untreated females in a ratio of 1:3 for 10 days (two complete estrous cycles)^15^. The presence of vaginal plugs and sperm in vaginal smears was checked to confirm mating. The following indices were calculated^16^:

Mating index = (number of males that mated, resulting in the vaginal plug or pregnant female/No. of males cohoused with females) × 100.

Fertility index = (number of males which sired a litter/No. Of males resulting vaginal plugs or pregnant females) × 100.

### Body and testicular weight and gonadosomatic index

Weight changes were continuously monitored and initial and final weights were recorded. At the end of the experiment (sacrifice day), testicular weights were recorded, and the gonadosomatic index (GSI) was calculated as: (testes weight / body weight) × 100^17^.

### Sperm analysis

The cauda epididymis was dissected and placed in a preheated Petri dish with 1 mL of saline solution at 37 °C. The sample was dissected with scalpels for 1 min and incubated at 37 °C for 15 min. Sperm motility, viability, count, and abnormalities were analyzed in at least 200 spermatozoa per animal. Motility was calculated by estimating the proportion of motile sperm. Viability was estimated by eosin‑nigrosin staining. Sperm abnormalities were categorized based on physical anomalies in the head, midpiece, and tail. For sperm count, 10 µL of diluted sample was placed in a counting chamber of a hemocytometer^18^.

### Comet assay (DNA Damage)

The comet assay was used to evaluate DNA damage as described by Singh et al.^19^. Epididymal cells were embedded in agarose layers on frosted slides and lysed at 4 °C to release DNA, then subjected to alkaline unwinding and electrophoresis (25 V, 300 mA, 20 min). Slides were neutralized, stained with ethidium bromide, and examined under a fluorescence microscope (400×). Fifty cells per animal were analyzed for tail length (measured from the nucleus to the tail edge) as an indicator of DNA damage. The percentage of damaged cells and mean tail length were statistically compared using the Mann‑Whitney test.

### Biochemical assays

Blood samples were collected from the retro-orbital sinus, allowed to clot for 10 min, and then centrifuged at 3000 rpm to obtain serum. Serum was analyzed for testosterone, follicle-stimulating hormone (FSH), and luteinizing hormone (LH) using commercial ELISA kits (Demeditec Diagnostics GmbH, Germany; Amersham, UK) according to the manufacturers’ protocols. Hormonal results were expressed as ng/mL for testosterone and mIU/mL for FSH and LH^20^. Total antioxidant capacity (TAC) was estimated by the ferric reducing antioxidant power (FRAP) method, where serum samples were incubated with freshly prepared FRAP reagent at 37 °C, and the absorbance of the formed blue complex was read at 595 nm^21–23^. Malondialdehyde (MDA), a marker of lipid peroxidation, was quantified using an HPLC-based method^24^. Catalase (CAT) activity was assayed spectrophotometrically according to a modified method by Hadwan et al.^25^.

### Histopathological examination

For each animal, the testis and epididymis were fixed in 10% neutral buffered formalin, and then processed to obtain paraffin sections of 5 μm thickness. Specimens were stained with hematoxylin and eosin (H&E)^26^ and examined by light microscope.

### Morphometric analysis

Morphometric evaluation was performed on H&E-stained testicular sections. Approximately 30 round or nearly round seminiferous tubules were randomly selected per animal and analyzed using ImageJ software (NIH, Bethesda, MD, USA) connected to an Olympus BX-40 microscope. Tubular diameters (longitudinal and transverse) were measured at 100× magnification and expressed in micrometers. Germinal epithelium height (measured from the basement membrane to the outermost spermatid layer) and luminal diameter were recorded. The mean diameter of testicular blood vessels was also determined^27^.

### Statistical analysis

Statistical analysis was conducted using ANOVA and Duncan’s Multiple Range test to assess differences among treatment means at a significance level of *p* < 0.05 (significant), *p* < 0.01 (highly significant), and *p* < 0.001 (very highly significant). Standard errors were calculated^28^. All analyses were conducted with SPSS software version 26 (IBM, Armonk, NY, USA). Bar charts were created using Microsoft Office 2019 (Redmond, WA, USA).

## Results

### LD_50_ determination

**Fig. 1:**
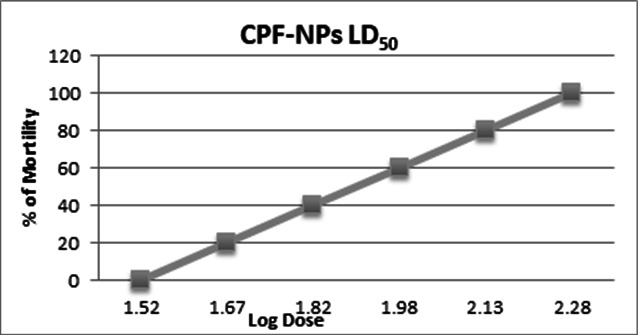
Graphical curve of LD_50_ of CPF-NE.

The LD_50_ for CPF-NE was assessed by the graphical method of Miller and Tainter^13^. The log dose corresponding to 50% mortality was 1.88, giving an LD_50_ of 75.6 mg/kg (Fig. 1; Table 1).

**Table 1:** Calculation of LD_50_ of CPF-NE according to Miller and Tainter (1944).

Group	Dose mg/kg	Dead	Survived	Dead %	Correction %	Log dose
1	190.8	10	0	100	99.7	2.28
2	134.95	8	2	80	80	2.13
**3**	**95.4**	**6**	**4**	**60**	**60**	**1.98**
**4**	**67.45**	**4**	**6**	**40**	**40**	**1.82**
**5**	**47.69**	**2**	**8**	**20**	**20**	**1.67**
**6**	**33.73**	**0**	**10**	**0**	**2.5**	**1.52**

0% of dead animals = 100 × 0.25/n.

100% of dead animals = 100 (n – 0.25/n).

### Clinical signs

Mice treated with CPF-NE showed dose-dependent cholinergic toxicity. Clinical manifestations included diarrhea, excessive salivation, muscle weakness, paralysis, and occasional loss of consciousness. These symptoms are typical of organophosphate poisoning.

### Reproductive performance

**Fig. 2:**
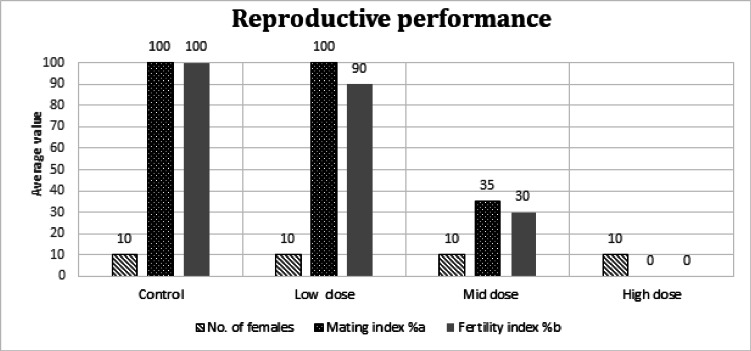
Reproductive performance in male albino mice orally treated with different doses of CPF-NE for 35 days.

Control males exhibited normal reproductive activity with 100% mating and fertility indices. No significant alterations were observed in the low-dose group. At the mid-dose, both indices markedly declined (*p* < 0.05). In the high-dose group, mating and fertility indices dropped to zero, indicating complete loss of reproductive capability (Fig. 2).

**a** No. Of males that mated, resulting in the vaginal plug or pregnant female/No. of males cohoused with females X100.

b No. Of males which sired a litter/No. Of males resulting vaginal plugs or pregnant females, 100.

### Body & testicular weights and gonadosomatic index (GSI)

CPF-NE caused significant reductions in body weight, testes weight, and GSI (*p* < 0.05 to *p* < 0.001). Body weight gain decreased from + 12% in controls to -4.69% in the high-dose group. Total testes weights were significantly reduced, and GSI decreased from 0.66 ± 0.02 (control) to 0.31 ± 0.02 (high dose) (Table 2).

**Table 2:** Final body weight, total testes weights and gonadosomatic index in male albino mice orally treated with different doses of CPF-NE for 35 days.

GROUPS	Final body weight (g)	Total testes weights (g)	gonadosomatic index (GSI)(%)
CONTROL	36.4 ± 0.3	0.24 ± 0.01	0.066 ± 0.02
Low dose(1/20)	35.4 ± 0.32	0.18 ± 0.01**	0.051 ± 0.02*
**Mid dose(1/10)**	33.7 ± 0.36**	0.14 ± 0.01**	0.034 ± 0.02*
**High dose(1/5)**	30.26 ± 0.38***	0.1 ± 0.004***	0.31 ± 0.02*
**F VALUE**	**59.45**	**81.8**	**55.53**

Data are expressed as a mean ± S.E. * Statistically significant compared to the control group (*P <* 0.05). ** Statistically highly significant compared to the control group (*P <* 0.01). *** Statistically very highly significant compared to the control group (*P <* 0.001).

### Sperm parameters

**Fig. 3:**
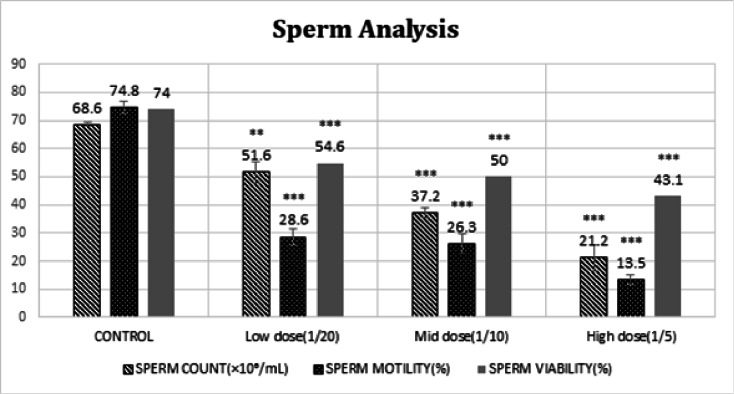
Sperm analysis of male albino mice orally treated with different doses of CPF-NE for 35 days. Data were expressed as a mean ± S.E. ** Statistically highly significant compared to the control group (*P <* 0.01). *** Statistically very highly significant compared to the control group (*P <* 0.001).

CPF-NE significantly decreased sperm quality. Motile sperm decreased from 74.8 ± 2.2% (control) to 28.6 ± 2.8%, 26.3 ± 3.6%, and 13.5 ± 1.7% in low, mid, and high doses, respectively (*p* < 0.001). Sperm viability declined from 74 ± 1.6% (control) to 54.6 ± 2.4%, 50 ± 1.9%, and 43.1 ± 1.7% (*p* < 0.001). Epididymal sperm concentration decreased from 68.6 ± 0.6 × 10⁶/mL (control) to 51.6 ± 3.7, 37.2 ± 1.6, and 21.2 ± 4.6 × 10⁶/mL (*p* < 0.001; Fig. 3).

### Sperm abnormalities

**Fig. 4:**
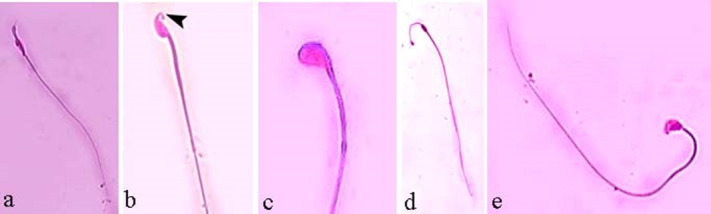
Sperm head abnormalities in male albino mice orally treated with different doses of CPF-NE for 35 days; (**a**) banana shaped with bent head, (**b**) hook with knobby appearance, (**c**) Amorphous head, (**d**) pin-like head, and (**e**) hookless head (×1000).

**Fig. 5:**
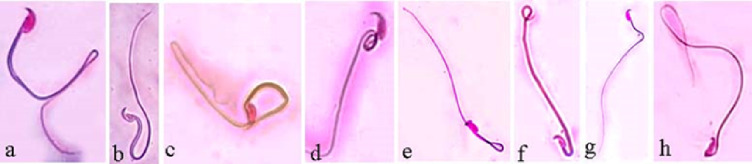
Sperm mid-piece and tail abnormalities in male albino mice orally treated with different doses of CPF-NE for 35 days; (**a**) bent tail, (b, e and g) bent mid-piece, (**c**) curved mid-piece, (**d**) coiled mid-piece, (f&h) coiled tail (×1000).

CPF-NE induced various head, midpiece, and tail abnormalities; including amorphous head, hook with knobby appearance, pin-shaped head, coiled midpiece, short tails, bent tails, and coiled tails (Tables 3 and 4; Figs. 4 and 5). Total abnormalities increased significantly from 15 ± 1 (control) to 39.8 ± 1.26, 59 ± 1.1, and 81.6 ± 1.45 in low, mid, and high doses (*p* < 0.05; Table 3).

**Table 3:** Total abnormalities of male albino mice orally treated with different doses of CPF-NE for 35 days.

GROUPS	Total abnormalities
CONTROL	15 ± 1
Low dose (1/20)	39.8 ± 1.26*
**Mid dose (1/10)**	59 ± 1.1*
**High dose (1/5)**	81.6 ± 1.45*
**F VALUE**	**542.1**

* Statistically significant compared to the control group (*P <* 0.05).

**Table 4:**
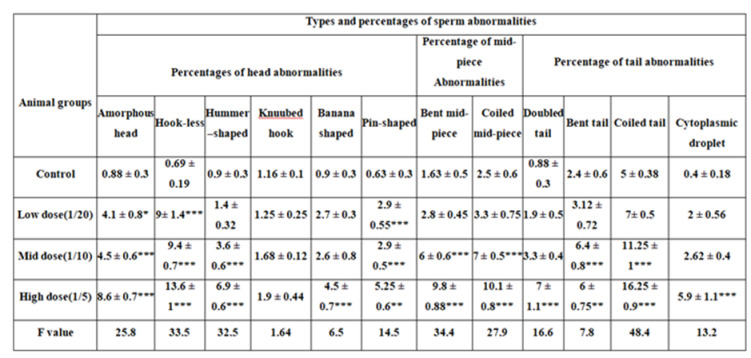
Sperm abnormalities in male albino mice orally treated with different doses of CPF-NE for 35 days.

Data were expressed as a mean ± S.E. * Statistically significant compared to the control group (*P <* 0.05). *** Statistically very highly significant compared to the control group (*P <* 0.001).

#### Comet assay (DNA Damage)

**Fig. 6:**
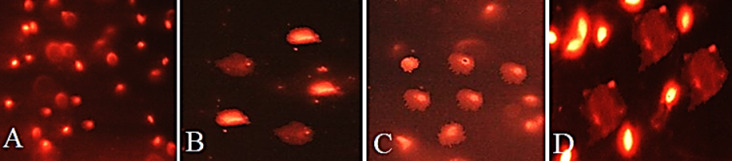
Photographs showing comet assay of male albino mice orally treated with different doses of CPF-NE for 35 days, (**A**): Control, (**B**): low dose 1\20LD50, (**C**) mid-dose 1\10 LD50, and (**D**) high dose 1\5 LD50.

Epididymal sperms showed significant DNA fragmentation in CPF-NE-treated groups. Tail DNA percentage and tail moment increased in a dose-dependent manner (*p* < 0.001; Fig. 6; Table 5).

**Table 5:** Comet assay of epididymal sperms of male albino mice orally treated with different doses of CPF-NE for 35 days, (A): Control, (B): low dose 1\20LD50, (C) mid-dose 1\10 LD50, and (D) high dose 1\5 LD50.

GROUPS	Comet %	Tail length (µm)	%DNA in tail	Tail moment (arbitrary units)
CONTROL	11.47 ± 0.18	4.88 ± 0.28	4.3 ± 0.37	0.32 ± 0.004
Low dose(1/20)	16.87 ± 0.08***	8.28 ± 0.56*	5.76 ± 0.14	0.61 ± 0.07**
**Mid dose(1/10)**	22.65 ± 0.23***	10.78 ± 0.08***	7.76 ± 0.2**	0.75 ± 0.09***
**High dose(1/5)**	29 ± 0.37***	17.02 ± 0.98***	14.35 ± 1.35***	0.84 ± 0.03***
**F VALUE**	**1009.56**	**77.32**	**39.15**	**15.27**

Data are expressed as a mean ± S.E. * Statistically significant compared to the control group (*P <* 0.05). ** Statistically highly significant compared to the control group (*P <* 0.01). *** Statistically very highly significant compared to the control group (*P <* 0.001).

### Hormonal analysis

**Fig. 7:**
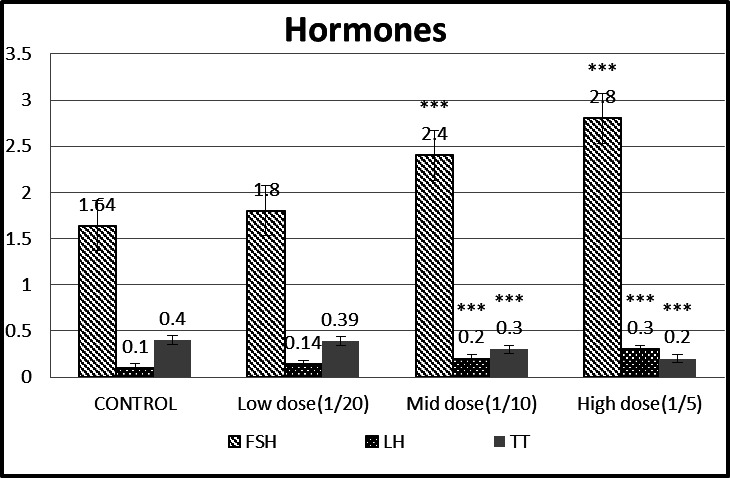
Hormonal analysis of male albino mice orally treated with different doses of CPF-NE for 35 days, FSH; follicular stimulating hormone(mIU/mL), LH; luteinizing hormone (mIU/mL), and TT; testosterone (ng/mL). Data were expressed by mean ± S.E. *** Statistically very highly significant compared to the control group (*P <* 0.001).

CPF-NE disrupted hormone levels. Total testosterone was significantly reduced in mid- and high-dose groups (control: 4.2 ± 0.3 ng/mL; high dose: 1.8 ± 0.2 ng/mL, *p* < 0.001). FSH and LH levels were significantly elevated in mid- and high-dose groups (control FSH: 2.1 ± 0.2 mIU/mL; high dose FSH: 5.4 ± 0.3 mIU/mL, *p* < 0.001; control LH: 1.5 ± 0.1 mIU/mL; high dose LH: 4.2 ± 0.2 mIU/mL, *p* < 0.001). No significant changes were observed in the low-dose group (Fig. 7).

#### Oxidative stress

**Fig. 8:**
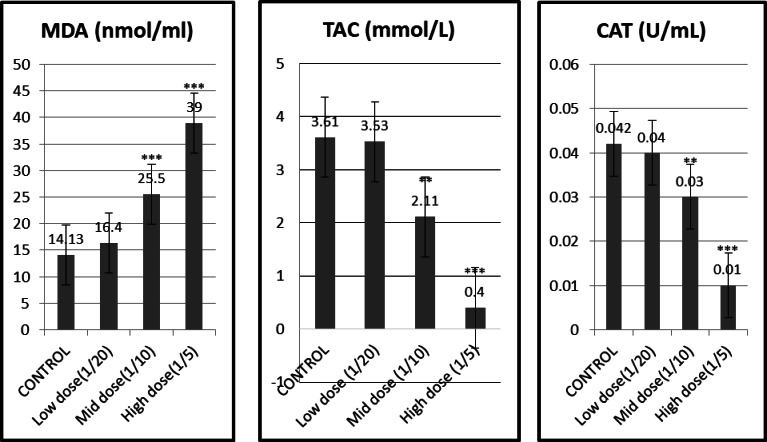
Histogram showing oxidative stress parameters of male albino mice orally treated with different doses of CPF-NE for 35 days, MDA: Malondialdehyde (nmol/mL), TAC: Total antioxidant capacity (mmol/L), CAT: Catalase (U/mL). Data were expressed by mean ± S.E. ** Statistically highly significant compared to the control group (*P <* 0.01). *** Statistically very highly significant compared to the control group (*P <* 0.001).

CPF-NE induced oxidative stress. Mid- and high-dose groups showed significantly increased MDA levels (control: 1.2 ± 0.1 nmol/mL; high dose: 3.8 ± 0.2 nmol/mL, *p* < 0.001). TAC was significantly decreased at mid- and high doses (control: 1.5 ± 0.1 mmol/L; high dose: 0.6 ± 0.1 mmol/L, *p* < 0.001). CAT activity also decreased significantly at mid- and high doses (control: 85.3 ± 3.2 U/mL; high dose: 32.1 ± 2.5 U/mL, *p* < 0.001) (Fig. 8).

#### Histological findings

**Fig. 9:**
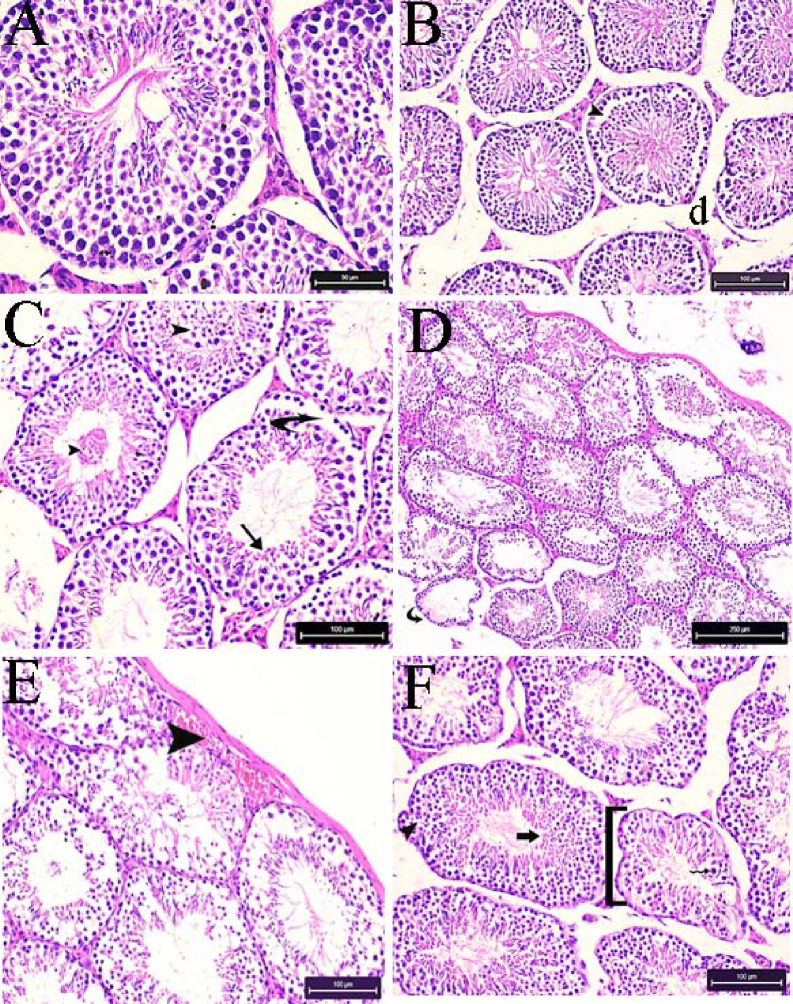
Photomicrographs of testicular histopathology (H&E) in an adult male mouse control group (**A**). Male albino mice were orally treated with various doses of CPF-NE for 35 days (**B-F**). B: Detached spermatogenic cell from the irregular basal lamina (arrow head) and dilated interstitial space with small groups of Leydig cells (d); C: Sloughing necrotic spermatocytes in tubular lumen (arrow head) and Cytoplasmic waste and debris (arrow); D: Spermatogenic arrest (bent arrow); E: Dilatation and congestion of blood vessels (arrow) and thickened tunica albugina; F:hyperplasia of spermatogenic cells( arrow head), irregular seminiferous tubules outlines (brackets), and Giant pyknotic spermatocyte (kinky arrow). Scale bars (50, 100,100, 250, 100, and 100 μm, respectively).

**Fig. 10:**
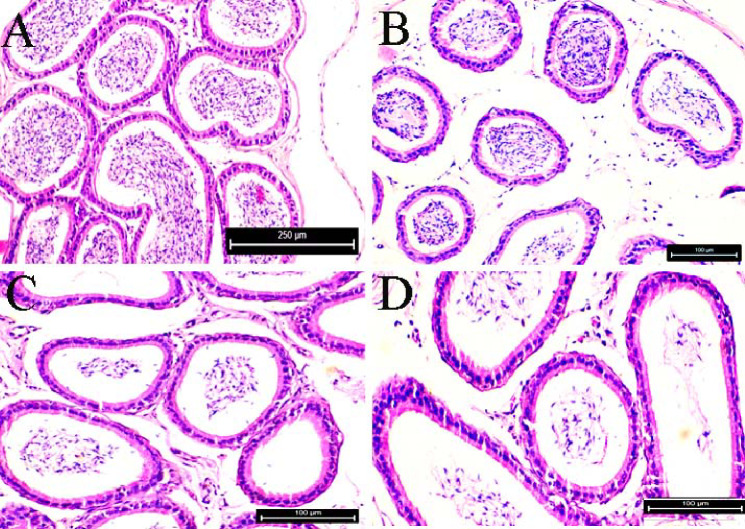
A photomicrograph of the cauda epididymis in adult male albino mice, stained with H&E, showing: A: control group, no pathological change, tubules have a thick, muscular covering. The lining is composed of tall, ciliated columnar cells. (B-D): Adult male albino mice orally treated with varying doses of CPF-NE for 35 days, (B): Moderate reduction in sperm number in the lumen, in (C&D): High reduction in sperm content in the epididymal lumen. Scale bars (250,100,100 and 100 μm, respectively).

Control mice showed normal testicular architecture with organized seminiferous tubules, intact germinal epithelium, and visible spermatozoa. In CPF-NE-treated mice, testicular damage was dose-dependent. Observed changes included tubular shrinkage, irregular basement membranes, separation of germinal epithelium from the basement membrane, pyknotic nuclei, vacuolation, sloughing of spermatogenic cells, and luminal cellular debris. Some tubules showed spermatogenic arrest with no mature sperm. Interstitial tissue exhibited vascular congestion and edema. The cauda epididymis showed a progressive, dose-dependent reduction in sperm content within the lumen (Figs. 9 and 10).

#### Morphometric analysis

CPF-NE caused significant structural changes. Longitudinal tubular diameter decreased from 206.15 ± 1.71 μm (control) to 138.31 ± 0.46 μm (high dose, *p* < 0.05). Transverse diameter decreased from 141.5 ± 1.64 μm to 92.57 ± 0.73 μm. Germinal epithelial height decreased from 36.77 ± 2.3 μm to 22.93 ± 0.6 μm (*p* < 0.05). Blood vessel diameter increased from 63.8 ± 0.6 μm to 77.58 ± 0.25 μm (*p* < 0.05). Tubular lumen diameter increased from 23.48 ± 1.1 μm to 36.08 ± 0.49 μm (*p* < 0.05). Tunica albuginea thickness showed no significant change (Table 6).

**Table 6:** Morphometric analysis of testes of male albino mice orally treated with different doses of CPF-NE for 35 days.

Parameters Groups	Tubular diameter (µm)		Epithelial height (µm)	Tubular lumen (µm)		Thickness of tunica Albuginea (µm)		Diameter of blood vessels (µm)
	L.S	T. S						
CONTROL	206.15 ± 1.71	141.5 ± 1.64	36.77 ± 2.3		23.48 ± 1.1		6.16 ± 0.62	63.8 ± 0.6
**Low dose (1/20)**	187.11 ± 3.0 8*	125.8 ± 1.92*	**31.94 ± 0.71**		28.01 ± 0.6*		**5.98 ± 0.07**	69.66 ± 0.59*
**Mid dose (1/10)**	147.91 ± 1.52*	111.32 ± 0.64*	28.62 ± 0.56*		34.03 ± 0.46*		**8.04 ± 0.08**	74.12 ± 0.46*
**High dose (1/5)**	138.31 ± 0.46*	92.57 ± 0.73*	22.93 ± 0.6*		36.08 ± 0.49*		4.36 ± 0.06*	77.58 ± 0.25*
**F VALUE**	**276.7**	**237.33**	**20.89**		**65.74**		**7.08**	**144.94**

* Statistically significant compared to the control group (*P <* 0.05).

## Discussion

The present study investigated the reproductive toxicity of CPF-NE in male albino mice. Mice treated with CPF-NE exhibited clear cholinergic toxicity, consistent with the well-known mechanism of CPF toxicity involving irreversible inhibition of acetylcholinesterase (AChE), leading to acetylcholine accumulation and overstimulation of cholinergic pathways. This resulted in excessive salivation, diarrhea, muscle weakness, and paralysis, with severity correlating with dose. These findings align with previous reports^29–34^.

CPF-NE exposure markedly reduced male fertility outcomes, including mating success and fertility indices. Our results are consistent with studies showing that CPF adversely affects male reproductive performance^10,35,36^. The decline in fertility index may be attributed to reduced sperm parameters, testicular histological alterations, and decreased testosterone levels^37^.

Decreased body weight in CPF-NE-treated mice agrees with previous observations^38–42^ and may reflect metabolic disturbances or gut microbiota alterations affecting nutrient absorption^23,29,44^. Testicular weight loss, a key indicator of testicular toxicity, has been linked to degenerative changes in seminiferous tubules and reduced spermatogenesis^1,7,40,45^.

CPF-NE caused significant deterioration in sperm parameters (count, motility, viability) and increased morphological abnormalities. This likely results from energy depletion due to inhibited oxidative phosphorylation and low ATP levels critical for sperm movement. Our findings align with previous studies^7,45,46^. Mechanistically, CPF induces oxidative stress and excessive ROS generation, disrupting androgen receptor-dependent pathways and triggering apoptosis in Leydig and Sertoli cells, thereby inhibiting spermatogenesis^1^.

The comet assay demonstrated CPF-NE-induced sperm DNA damage, consistent with genotoxicity reports in insects, fish, and mammals^47–52^. CPF generates excess free radicals leading to oxidative DNA strand breakage, a primary mechanism of reproductive toxicity.

CPF-NE significantly reduced serum testosterone while elevating FSH and LH, indicating disruption of the hypothalamic-pituitary-gonadal (HPG) axis. Elevated gonadotropins likely represent a compensatory response to decreased testosterone and impaired Leydig cell function^40–42,53^. Similar hormonal imbalances have been reported following CPF or organophosphate exposure^38,43^. AChE inhibition by CPF may also affect HPG axis regulation^54,55^. Discrepancies in gonadotropin levels across studies may reflect differences in dose or exposure duration^56^.

Our study showed increased malondialdehyde (MDA) and decreased total antioxidant capacity (TAC) and catalase (CAT) activity, corroborating previous findings^9,57,58^. Elevated MDA indicates lipid peroxidation, while reduced TAC and CAT reflect depletion of endogenous antioxidant systems, contributing to reproductive toxicity.

Histological examination of testes from CPF-NE-treated mice revealed dose-dependent structural damage, including seminiferous tubule shrinkage, irregular basement membranes, germinal epithelial detachment, pyknotic nuclei, vacuolation, sloughing of spermatogenic cells, and luminal debris. Some tubules showed spermatogenic arrest with no mature sperm. Interstitial vascular congestion and edema were also observed. These findings are consistent with CPF-induced tubular degeneration reported in rodents^18,59^. Morphometric analysis confirmed reduced tubular diameters, decreased germinal epithelial height, increased luminal diameter, and enlarged blood vessels, indicating compromised structural integrity. Similar degenerative changes have been described^18,60,61^. Oxidative stress-induced ROS production leads to lipid peroxidation and DNA damage, increasing apoptosis in germline cells^62^. Disruption of the HPG axis and reduced testosterone further impair spermatogenesis^63^.

In summary, CPF-NE adversely affects male fertility through impaired reproductive performance, testicular and sperm developmental toxicity, increased DNA fragmentation, hormonal disruption, and oxidative stress. The cumulative evidence indicates that oxidative stress is a primary mediator of CPF-NE-induced reproductive toxicity. While direct comparison with conventional CPF was not performed, the severity of effects at relatively low doses suggests that the nanoformulation may enhance bioavailability and toxicity. Future studies should include chronic exposure models, occupational risk assessment, and regulatory toxicology testing for nano-pesticides.

## Conclusion

Our current study demonstrates that CPF-NE has notable dose-related reproductive toxicity in male mice. Exposure to CPF-NE led to negative effects on reproductive performance, sperm attributes, and testicular histoarchitecture, along with induction of oxidative stress, DNA damage, and endocrine disruption. Perturbation of the hypothalamic-pituitary-gonadal axis and oxidative stress in the germinal epithelium appear to be key interrelated factors in CPF-NE-induced infertility. These results suggest a potential for enhanced reproductive toxicity from the nanoformulation, though direct comparative studies with non-nano CPF are required. Future research should include chronic exposure models, occupational risk assessment, and regulatory toxicology testing for nano-pesticides.

## Limitations

The mechanistic pathway is limited due to lack of molecular pathway analysis, long-term exposure models, and comparison with non-nano CPF. Additional studies are necessary to better define dose–response relationships and chronic reproductive hazards.

## Data Availability

The data supporting the findings of this study are available from the corresponding author upon reasonable request.

## Abbreviations

CPF: Chlorpyrifos
FSH: Follicular Stimulating Hormone
LH: Lutiniing Hormones
TAC: Total Antioxidant Capacity
MDA: Malondialdehyde
CAT: Catalase
Ops: Organophosphates
GSI: Gonadosomatic Index
CPF-NE: Chlorpyrifos nano-emulsion
Ache: Acetylcholinesterase
ROS: Reactive Oxygen Species
HPG: Hypothalamic–Pituitary–Gonadal
